# Effects of genetically predicted posttraumatic stress disorder on autoimmune phenotypes

**DOI:** 10.1038/s41398-024-02869-0

**Published:** 2024-04-01

**Authors:** Adam X. Maihofer, Andrew Ratanatharathorn, Sian M. J. Hemmings, Karen H. Costenbader, Vasiliki Michopoulos, Renato Polimanti, Alex O. Rothbaum, Soraya Seedat, Elizabeth A. Mikita, CHARGE Inflammation Working Group, Psychiatric Genomics Consortium PTSD Working Group, Alicia K. Smith, Rany M. Salem, Richard A. Shaffer, Tianying Wu, Jonathan Sebat, Kerry J. Ressler, Murray B. Stein, Karestan C. Koenen, Erika J. Wolf, Jennifer A. Sumner, Caroline M. Nievergelt

**Affiliations:** 1 Department of Psychiatry, University of California San Diego, La Jolla, CA USA; 2 Veterans Affairs San Diego Healthcare System, Center of Excellence for Stress and Mental Health, San Diego, CA USA; 3 Herbert Wertheim School of Public Health and Human Longevity Science, University of California San Diego, La Jolla, CA USA; 4 Veterans Affairs San Diego Healthcare System, San Diego, CA USA; 5 Department of Epidemiology, Harvard T. H. Chan School of Public Health, Boston, MA USA; 6 Department of Epidemiology, Columbia University Mailman School of Public Health, New York, NY USA; 7 Department of Psychiatry, Faculty of Medicine and Health Sciences, Stellenbosch University, Cape Town, Western Cape South Africa; 8 South African Medical Research Council/Genomics of Brain Disorders Research Unit, Faculty of Medicine and Health Sciences, Stellenbosch University, Cape Town, South Africa; 9 Division of Rheumatology, Inflammation and Immunity, Department of Medicine, Brigham and Women’s Hospital, Harvard Medical School, Boston, MA USA; 10 Department of Psychiatry and Behavioral Sciences, Emory University, Atlanta, GA USA; 11 VA Connecticut Healthcare Center, West Haven, CT USA; 12 Department of Psychiatry, Yale University School of Medicine, New Haven, CT USA; 13 Department of Research and Outcomes, Skyland Trail, Atlanta, GA USA; 14 Department of Gynecology and Obstetrics, Emory University, Atlanta, GA USA; 15 Department of Epidemiology and Health Sciences, Naval Health Research Center, San Diego, CA USA; 16 Division of Epidemiology and Biostatistics, School of Public Health, San Diego State University, San Diego, CA USA; 17 Moores Cancer Center, University of California, San Diego, San Diego, CA USA; 18 Cold Spring Harbor Laboratory, Cold Spring Harbor, NY USA; 19 Department of Cellular and Molecular Medicine, University of California San Diego, La Jolla, CA USA; 20 Department of Psychiatry, Harvard Medical School, Boston, MA USA; 21 McLean Hospital, Belmont, MA USA; 22 VA Boston Healthcare System, National Center for PTSD, Boston, MA USA; 23 Department of Psychiatry, Boston University Chobanian & Avedisian School of Medicine, Boston, MA USA; 24 Department of Psychology, University of California Los Angeles, Los Angeles, CA USA

**Keywords:** Genetics, Psychiatric disorders, Diagnostic markers

## Abstract

Observational studies suggest that posttraumatic stress disorder (PTSD) increases risk for various autoimmune diseases. Insights into shared biology and causal relationships between these diseases may inform intervention approaches to PTSD and co-morbid autoimmune conditions. We investigated the shared genetic contributions and causal relationships between PTSD, 18 autoimmune diseases, and 3 immune/inflammatory biomarkers. Univariate MiXeR was used to contrast the genetic architectures of phenotypes. Genetic correlations were estimated using linkage disequilibrium score regression. Bi-directional, two-sample Mendelian randomization (MR) was performed using independent, genome-wide significant single nucleotide polymorphisms; inverse variance weighted and weighted median MR estimates were evaluated. Sensitivity analyses for uncorrelated (MR PRESSO) and correlated horizontal pleiotropy (CAUSE) were also performed. PTSD was considerably more polygenic (10,863 influential variants) than autoimmune diseases (median 255 influential variants). However, PTSD evidenced significant genetic correlation with nine autoimmune diseases and three inflammatory biomarkers. PTSD had putative causal effects on autoimmune thyroid disease (*p* = 0.00009) and C-reactive protein (CRP) (*p* = 4.3 × 10^−7^). Inferences were not substantially altered by sensitivity analyses. Additionally, the PTSD-autoimmune thyroid disease association remained significant in multivariable MR analysis adjusted for genetically predicted inflammatory biomarkers as potential mechanistic pathway variables. No autoimmune disease had a significant causal effect on PTSD (all *p* values > 0.05). Although causal effect models were supported for associations of PTSD with CRP, shared pleiotropy was adequate to explain a putative causal effect of CRP on PTSD (*p* = 0.18). In summary, our results suggest a significant genetic overlap between PTSD, autoimmune diseases, and biomarkers of inflammation. PTSD has a putative causal effect on autoimmune thyroid disease, consistent with existing epidemiologic evidence. A previously reported causal effect of CRP on PTSD is potentially confounded by shared genetics. Together, results highlight the nuanced links between PTSD, autoimmune disorders, and associated inflammatory signatures, and suggest the importance of targeting related pathways to protect against disease and disability.

## Introduction

Posttraumatic stress disorder (PTSD), characterized by intrusive memories of traumatic experiences, avoidance of trauma-related stimuli, negative changes in thinking and mood, and high levels of arousal, is a debilitating psychiatric illness that can develop in response to exposure to trauma [1]. The burden of PTSD on individuals and society is extensive [2], with numerous emotional, interpersonal, and socioeconomic consequences [3–5]. Moreover, PTSD is also associated with numerous physical health outcomes [6], notably including a variety of health conditions and diseases related to immune dysregulation and inflammation [7, 8]. Longitudinal research has demonstrated PTSD to be associated with a heightened incidence of autoimmune diseases, including diseases of the endocrine, skin, nervous, digestive systems, connective tissues, inflammatory arthritis, and vasculitis [7–14]. Shared biological mechanisms related to immune dysregulation are thought to be a primary factor linking PTSD and autoimmune diseases, as studies of peripheral inflammatory biomarkers, the epigenome, transcriptome, and common genetic variation all support the notion of immune dysregulation being involved in PTSD pathophysiology [15–17].

While longitudinal observational studies suggest that PTSD precedes the development of autoimmune diseases, causal relationships have yet to be delineated [17, 18]. Insights into whether PTSD and a variety of autoimmune diseases are causally related may enhance biological understanding of PTSD and its sequelae, as well as inform intervention approaches for PTSD and co-morbid autoimmune diseases [17, 19, 20]. In addition to directly assessing the relationship between PTSD and autoimmune diseases, it is also beneficial to evaluate the relationship between PTSD and biomarkers of inflammation, which can be the first non-specific sign of an autoimmune disorder that prompts further evaluation [21]. Moreover, as inflammation underlies PTSD and autoimmune disease, it is of interest to determine whether inflammation confounds or mediates their relationship. Therefore, multiple causal questions need to be investigated: (1) if PTSD gives rise to autoimmune diseases or vice versa; (2) if this relationship is bi-directional; (3) if shared underlying pathology is a common cause (confounder) between PTSD and autoimmune disease; (4) if inflammation mediates or confounds the relationship between PTSD and autoimmune disease.

Two-sample Mendelian randomization (MR) [22] analyses can be utilized to test these questions. MR uses genetic variants as instrumental variables (i.e., presumedly unconfounded stand-in variables) for the phenotypes of interest, leveraging the random assignment of alleles at conception to produce an analysis akin to a randomized experiment. Prior MR analyses identified putative causal associations between psychiatric disorders and autoimmune diseases, but none have been identified for PTSD thus far [23, 24]. This may be due to lack of power: the instrumental variables selected were significant SNPs identified by large-scale genome-wide association studies (GWAS) [18], yet only a few significant loci were identified by prior PTSD GWAS [25]. However, with respect to the context of PTSD and inflammation, an MR study of PTSD and the inflammatory biomarker C-reactive protein (CRP) found significant evidence of a bidirectional causal association [26].

In this study, we expanded upon prior efforts by comprehensively investigating the shared genetic contributions and causal relationships between PTSD and a range of autoimmune diseases using well-powered GWAS summary statistics, including from a substantially larger PTSD GWAS [27] than previously utilized [25]. We contrasted the genetic architectures of PTSD, 18 autoimmune diseases, and 3 non-specific immune/inflammatory biomarkers, and investigated their genetic overlap. We used bidirectional two-sample MR to test causal hypotheses for PTSD, autoimmune disease, and immune/inflammatory biomarkers. We also conducted several sensitivity analyses to evaluate the robustness of our findings given the potential confounding effects of horizontal pleiotropy and reverse causation. Finally, for autoimmune diseases with significant MR findings, we applied multivariable MR analysis that adjusted for immune/inflammatory biomarkers. Under the assumption that inflammatory biomarkers serve as measures of systemic inflammation [28], the difference between multivariable and standard MR estimates would indicate the contribution of a general inflammatory signature (either as a mediator or confounder) to the relationship between PTSD and the autoimmune disease.

## Methods

### GWAS summary data collection and curation

We searched for GWAS of all phenotypes that were featured in a pair of recent, comprehensive studies of the relationship between PTSD and autoimmune diseases [8] and related immune/inflammatory biomarkers [29]. In total, 46 different phenotypes were considered (40 autoimmune diseases and 6 biomarkers) for investigation (Supplementary Table 1). From March 14–21_,_ 2023, we searched the NHGRI-EBI GWAS Catalog [30] and Google Scholar for GWAS of these phenotypes. Search criteria included [phenotype name] + GWAS. GWAS had been performed in 41 of the 46 phenotypes, making for 51 results retrieved (sometimes multiple GWAS had been performed for a given phenotype). When multiple GWAS were available for a given phenotype, we selected for investigation the GWAS with the largest number of case samples; it was generally that these GWAS were more recent and had included all samples from smaller previously reported GWAS of the same phenotype. Single nucleotide polymorphism (SNP)-level summary statistics were obtained from online publicly available data repositories or, if not available online, by request from study authors. Study authors were contacted for participation. If not provided a response within 1 month, authors were contacted a second time. Phenotypes were excluded if authors did not respond. Upon acquisition of GWAS summary data, to be included in our analysis, the data needed to contain rsID, the allele coded as the risk allele and the allele coded as the non-risk allele, effect sizes, corresponding standard errors, and *p* values. For statistical power reasons, only GWAS with at least one significant SNP instrument were included in our analysis. To prevent confounding due to ancestral differences, we only included GWAS of individuals of European ancestry. After these criteria were applied, 21 phenotypes were deemed suitable for analyses in this investigation (Supplementary Fig. 1). A complete list of GWAS identified for usage, along with inclusion/exclusion information, are provided in Supplementary Table 1.

### PGC-PTSD GWAS

The PGC-PTSD Freeze 3 European ancestry GWAS contains *N* = 1,222,882 participants (137,136 cases and 1,085,746 controls) from 88 studies [27]. Studies included civilian and military populations. PTSD was assessed with clinician-administered or self-report instruments or via ICD code derivation. Genotyping was conducted on Illumina or Affymetrix arrays. Standard quality control procedures were applied to genotype data. All datasets were imputed based on a population-suitable reference panel. GWAS were performed within European ancestry participants, adjusting for 5–10 principal components calculated within-sample. Sample size-weighted meta-analysis of GWAS summary data was conducted in METAL. Batches of contributing studies were compared for genetic overlap, where genetic correlation (*r*_*g*_) > 0.8 between all tested pairs, indicating excellent genetic overlap, despite heterogeneity in populations, genotyping, and methods of PTSD assessment. The *r*_*g*_ between male and female subsets was 0.95, thus sex-stratified analyses were not evaluated here.

### Phenotypes under investigation

After curating data, our phenotype collection included PTSD (obtained from the PGC-PTSD Freeze 3 GWAS [27]); 3 inflammatory/immune biomarkers: CRP [31], interleukin-6 (IL-6) [32], and white blood cell count (WBC) [33]; and 19 autoimmune diseases: primary adrenal insufficiency (Addison’s disease) [34], autoimmune thyroid disease [35], celiac disease [36], Crohn’s disease [37], eosinophilic granulomatosis with polyangiitis (Churg-Strauss syndrome) [38], mucocutaneous lymph node syndrome (Kawasaki disease) [39], multiple sclerosis [40], myasthenia gravis [41], neuromyelitis optica spectrum disorder [42], pernicious anemia [43], primary biliary cholangitis [44], psoriasis [45], rheumatoid arthritis [46], systemic lupus erythematosus [47], systemic sclerosis [48], type 1 diabetes [49], ulcerative colitis [50], and vitiligo [51] (Supplementary Table 1).

### Heritability and genetic overlap analyses

SNP based heritability (*h*^2^_SNP_) and *r*_*g*_ of phenotypes were estimated using linkage disequilibrium (LD) score regression (LDSC) [52]. LD scores calculated within 1000 Genomes Phase 3 European populations [53] were used for the input. Analyses were limited to HapMap 3 SNPs. For genetic correlation analyses, the major histocompatibility complex (MHC) region was excluded (hg19 coordinates: chromosome 6: 26–34 million base pairs). For binary traits, *h*^2^_SNP_ is reported on the liability scale adjusted to the population prevalence. *h*^2^_SNP_ is proportional to the product of two subcomponents: the proportion of non-null SNPs (polygenicity) and variance of effect sizes of non-null SNPs (discoverability). By estimating these subcomponents for each phenotype, it is possible to identify nuances that further clarify their genetic architectures, such as whether heritability is the result of a few variants with strong effects or many variants with weak effects. We used univariate MiXeR [54] (version 1.3) to estimate the polygenicity and discoverability of all phenotypes. Using these results, the polygenic overlap between phenotypes was estimated using bivariate MiXeR [55]. We used the default settings and the supplied 1000 Genomes European ancestry LD reference panel. For interpretability, rather than directly reporting polygenicity outputs from MiXeR, we reported the number of influential variants necessary to explain 90% of *h*^2^_SNP_ (calculated as polygenicity ✕ constant).

### MR analyses

The TwoSampleMR R package [56] was used to perform two-sample bidirectional MR. Effect allele coding was harmonized across phenotypes using the harmonise_data function. Strand ambiguous SNPs were excluded. To avoid weak instrument bias, genetic instruments were constructed using genome-wide significant SNPs. Genome-wide significant SNPs were LD clumped (*r*^2^ ≤ 0.001 in 1000 Genomes Phase 3 European data [53], over a 10 megabase window) to ensure independence. SNPs within two highly pleiotropic regions, the MHC region [57] (hg19 coordinates: Chromosome 6, 28,477,797–33,448,354 base pairs) and 17q21.31 region inversion (hg19 coordinates: Chromosome 17, 40,928,986–42,139,672 base pairs) were excluded, with a 3 megabase buffer added to ensure markers in LD were also removed. The primary MR analysis was conducted using the inverse variance weighted (IVW) estimator with multiplicative random effects. Additional MR analysis was performed using weighted median (WM) [58] estimators. Pearson correlations between IVW and WM estimates were estimated. To account for the multiple testing burden, Bonferroni correction was applied within each estimator, such that significance was declared if *p* < 0.05/21. As in prior research [28], multivariable MR analysis was performed using CRP, IL-6, and WBC as covariates to statistically control for non-specific markers of systemic inflammation that could confound or mediate associations of PTSD with autoimmune disease, using the MendelianRandomization R package [59]. These multivariable MR analyses were conducted for autoimmune disease phenotypes with significant MR findings. Owing to instrumental variable loss due to incomplete summary data overlap, immune/inflammatory biomarkers were modeled one at a time rather than together in a joint model.

### MR sensitivity analyses

Several sensitivity analyses were conducted to assess the robustness of findings. A substantial fraction of the heritability of autoimmune diseases is explained by a limited set of risk variation with large effect sizes. This lends to the possibility of reverse causation if these variants are also included in genetically predicted PTSD. Accordingly, we performed a sensitivity analysis where regions that were genome-wide significant in autoimmune GWAS (±3 megabases) were removed from genetically predicted PTSD. MR PRESSO [60] was used to identify heterogeneity (global test) and outliers (outlier test), and to determine if the outlier-adjusted IVW estimate was significantly different from the unadjusted. To evaluate if our associations could be explained by correlated horizontal pleiotropy (defined as genes influencing a third factor, which in turn has pleiotropic effects on the exposure and outcome), we used the CAUSE method [61]. This method fits a series of nested models: a “null” model where only uncorrelated horizontal pleiotropy (defined as direct effects of genes on the outcome with net zero effect) is modeled (parameter *q*), a “sharing” model where an additional parameter (parameter eta) is fit to account for correlated horizontal pleiotropy, and a “causal” model where a causal effect parameter (parameter gamma) is fit in addition to the sharing parameter. To test the hypothesis that a causal model explained the relationship better than a sharing model, the causal and sharing model fits were compared using the difference in the expected log pointwise posterior density. Specifically, if the causal model fits better than the sharing model, this implies that the additional complexity needed to model a causal effect is justified, and thus is evidence that data are consistent with a causal effect. If, however, there is not significant evidence that the causal model fits better than the sharing model, this implies that shared pleiotropy alone is sufficient to explain the observed association. To account for potential biases related to sample overlap [62], we repeated IVW MR analysis of significant findings with the UKBB sample data removed from the PTSD GWAS.

## Results

After filtering criteria, we assembled a collection of GWAS summary statistics for PTSD, 18 autoimmune diseases, and 3 biomarkers (Supplementary Table 1 and Supplementary Fig. 1). The PTSD GWAS included 1,222,822 participants (including 137,136 PTSD cases). The median autoimmune disease GWAS sample size was 38,078, and ranged from 1459 (neuromyelitis optica spectrum disorder) to 755,406 (autoimmune thyroid disease). The biomarker GWAS sample sizes were 52,654 (IL-6), 363,000 (CRP), and 562,243 (WBC).

### Genetic architecture of PTSD and immune-related phenotypes

**Fig. 1:**
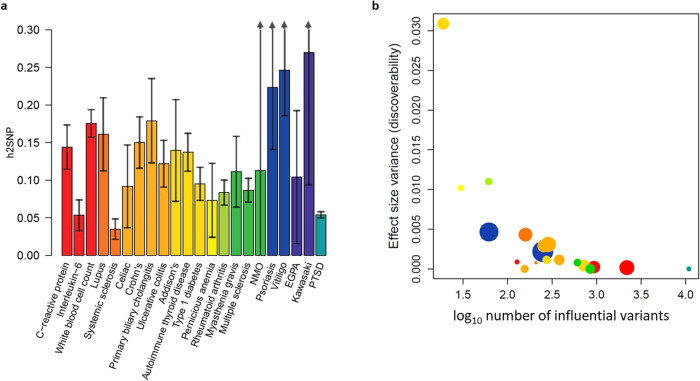
Genetic architectures of phenotypes evaluated. **a** SNP based heritability of assessed phenotypes. SNP based heritability estimates for autoimmune diseases are provided on the liability scale, assuming disease specific prevalence. Phenotypes are colored by category. Black bars ends indicate 95% confidence intervals. Confidence intervals extending beyond the plot range are indicated with arrows. **b** Polygenicity and discoverability components of heritability. The *x*-axis depicts the number of influential variants necessary to explain 90% of SNP based heritability (polygenicity ✕ constant). The *y*-axis depicts the discoverability of the phenotype. Circle sizes indicate relative SNP based heritability values. NMO Neuromyelitis Optica Spectrum Disorder, EGPA Eosinophilic granulomatosis with polyangiitis.

The *h*^2^_SNP_ of PTSD was 5.3% (on the observed scale). Estimates of *h*^2^_SNP_ varied across autoimmune diseases, ranging from 3.5% (systemic sclerosis) to 27% (Kawasaki disease) (Fig. 1a). The *h*^2^_SNP_ estimates of the three biomarkers were 5.3% (IL-6), 14.3% (CRP), and 17.5% (WBC). We separated *h*^2^_SNP_ into its polygenicity and discoverability subcomponents (Fig. 1b and Supplementary Table 2). PTSD was the most polygenic (*N* causal SNPs = 10,863, SE = 377) and least discoverable phenotype. Across autoimmune diseases, the estimated number of causal SNPs ranged from 18 (Addison’s disease) to 721 (autoimmune thyroid disease). For the three biomarkers, the estimated number of causal SNPs were 127 (IL-6), 934 (CRP), and 2187 (WBC).

**Fig. 2:**
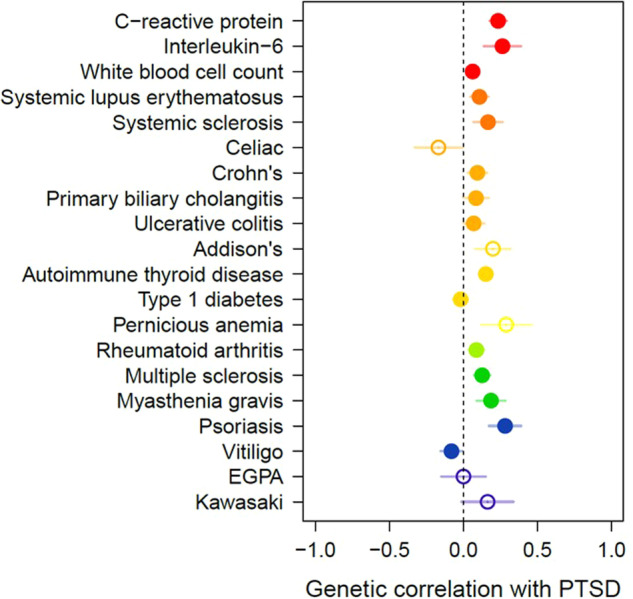
Genetic correlations between PTSD and immune-related phenotypes. Genetic correlations (*r*_*g*_) are indicated by circles that are drawn along the *x* axis. Phenotypes are colored by domain. Hollow circles indicate SNP based heritability (*h*^2^_SNP_) *z*-score <4 in the immune-related phenotype GWAS (*r*_*g*_ estimates may be unreliable). The dotted vertical bar indicates the point of zero correlation. EGPA eosinophilic granulomatosis with polyangiitis.

Previous reports [23] of psychiatric disorders and autoimmune diseases have used genetic correlation models to quantify their genetic overlap, and thus we first used this same approach to examine their genetic association. Under a genetic correlation model, PTSD was significantly positively correlated with nine autoimmune diseases and all three biomarkers after Bonferroni correction (Fig. 2 and Supplementary Table 3). We also examined expanded models of genetic overlap between PTSD and all phenotypes (Bivariate MiXeR). These models suggested that for 10 phenotypes examined, genetic overlap was more complex than what could be summarized by *r*_*g*_ alone (AIC values > 0). Most often, this meant that a substantial fraction of autoimmune disease/biomarker variants were influential on PTSD, and that the *r*_*g*_ estimated only among the reduced set of predicted shared variants was stronger than *r*_*g*_ estimated from all variants (Supplementary Table 4).

### Causal associations between PTSD and immune-related phenotypes

**Fig. 3:**
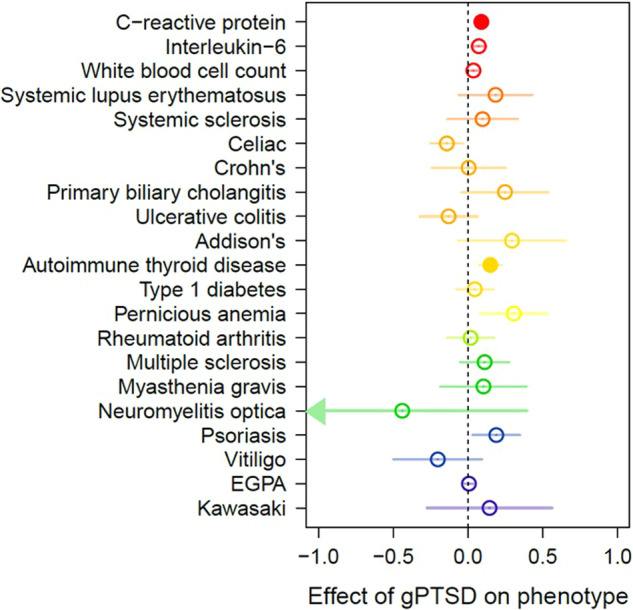
Putative causal effects of PTSD. Causal effects of PTSD on immune-related phenotypes, as estimated by the genetically determined PTSD instrument (gPTSD), are indicated by circles that are drawn along the *x* axis. Phenotypes are colored by domain. Hollow circles indicate non-significance (*p* > 0.0025). The dotted vertical bar indicates the point of zero effect. Confidence intervals extending beyond the plot range are indicated with arrows. EGPA eosinophilic granulomatosis with polyangiitis.

Genetically predicted PTSD (gPTSD) was estimated using 62 LD-independent genome-wide significant risk loci. Under the primary IVW analysis method, gPTSD was significantly positively associated with risk of autoimmune thyroid disease (beta = 0.150, SE = 0.018, *p* = 0.0001) and CRP (beta = 0.090, SE = 0.018, *p* = 4.3 × 10^−7^) after Bonferroni correction (Fig. 3 and Table 1). Under the WM analysis, gPTSD was significantly associated with elevated levels of CRP (beta WM = 0.071, SE = 0.015, *p* = 1.4 × 10^−6^) and WBC (beta = 0.036, SE = 0.011, *p* = 0.002). gPTSD effect size estimates were broadly similar between IVW and WM methods, with a 91% correlation between them. Several phenotypes (Crohn’s disease, ulcerative colitis, rheumatoid arthritis, multiple sclerosis, vitiligo, CRP, and WBC) indicated significant heterogeneity of SNP instruments (IVW heterogeneity statistic *p* < 0.05/21), indicative of horizontal pleiotropy. Similarly, the global test in MR PRESSO indicated the presence of outliers for these phenotypes. However, the gPTSD effect estimates were not significantly different for any phenotype after MR PRESSO outlier removal (all MR PRESSO distortion test *p* > 0.05; Supplementary Table 5).

**Table 1:** Mendelian randomization analysis of effects of genetically predicted PTSD on investigated phenotypes.

		Inverse variance weighted			IVW Het.	Weighted median		
Phenotype	*N* SNPs	Beta	SE	*p*^a^	*Q* *p*	Beta	SE	*p*^a^
Connective tissues								
Systemic lupus erythematosus	62	0.185	0.126	0.140	0.07	0.092	0.162	0.572
Systemic sclerosis	58	0.098	0.120	0.415	0.06	0.096	0.159	0.547
Digestive system								
Celiac	57	−0.141	0.055	0.011	0.03	−0.136	0.070	0.051
Crohn’s	61	0.005	0.126	0.968	**5.0E−08**	0.066	0.128	0.605
Primary biliary cholangitis	55	0.248	0.148	0.093	0.02	0.243	0.182	0.181
Ulcerative colitis	62	−0.129	0.099	0.194	**8.5E−04**	−0.117	0.113	0.300
Endocrine								
Addison’s	62	0.295	0.183	0.107	0.25	0.495	0.255	0.052
Autoimmune thyroid disease	62	0.150	0.038	**8.7E−05**	0.02	0.120	0.049	0.015
Type 1 diabetes	62	0.046	0.065	0.483	0.01	0.100	0.081	0.216
Hematological								
Pernicious anemia	62	0.307	0.115	0.008	0.57	0.299	0.162	0.065
Inflammatory arthritis								
Rheumatoid arthritis	62	0.018	0.081	0.824	**1.0E−07**	0.120	0.083	0.147
Nervous system								
Multiple sclerosis	60	0.112	0.083	0.177	**3.7E−05**	−0.023	0.087	0.795
Myasthenia gravis	60	0.104	0.147	0.478	0.17	0.015	0.199	0.939
Neuromyelitis optica	62	−0.439	0.427	0.303	0.17	−0.455	0.574	0.428
Skin								
Psoriasis	61	0.190	0.081	0.019	0.19	0.232	0.115	0.044
Vitiligo	57	−0.201	0.151	0.185	**1.5E−05**	−0.229	0.175	0.189
Vasculitis								
Churg Strauss	61	0.007	0.015	0.620	0.40	−0.004	0.021	0.843
Kawasaki	60	0.145	0.214	0.498	0.16	0.324	0.302	0.283
Biomarkers								
C-Reactive protein	61	0.090	0.018	**4.3E−07**	**1.8E−25**	0.071	0.015	**1.38E−06**
Interleukin-6	54	0.073	0.025	0.004	0.68	0.056	0.037	0.137
White blood cell count	56	0.037	0.014	0.007	**9.9E−20**	0.036	0.011	**0.002**

*N SNPs* number of single nucleotide polymorphisms used as instruments, *IVW Het* inverse variance weighted meta-analysis heterogeneity test.

^a^Bolded where Bonferroni significant.

As a sensitivity analysis for reverse causation, any regions that were significant in the target phenotype GWAS (±3 megabases) were not used as SNP instruments in gPTSD. The gPTSD associations with autoimmune thyroid disease (*N* SNP instruments = 52, IVW beta = 0.122, SE = 0.037, *p* = 0.001) and CRP (*N* SNP instruments = 40, IVW beta = 0.084, SE = 0.015, *p* = 1 × 10^−7^) remained significant. We also performed MR in the opposite causal effect direction, examining the association of genetically predicted phenotypes with PTSD (Supplementary Table 6). We did not identify any significant associations.

As a sensitivity analysis for sample overlap, we removed the UK BioBank data from the PTSD GWAS, as it represented the main source of sample overlap. The IVW effect estimates of PTSD on autoimmune thyroid disease (*N* = 38 instruments, beta = 0.11, SE = 0.04, *p* = 0.01) and CRP (*N* = 38 instruments; beta = 0.069, SE = 0.02, *p* = 0.001) were comparable in magnitude to the original estimates.

### Influence of third variables on putative causal effect estimates

Systemic inflammation may link PTSD to autoimmune disease. To explore this pathway, we performed multivariable MR analysis that adjusted for genetically predicted CRP, IL-6, and WBC as surrogate measures of systemic inflammation. In each multivariable model, inference of the association between gPTSD and autoimmune thyroid disease was not substantially altered (CRP adjusted gPTSD IVW beta = 0.162, SE = 0.047, *p* = 0.0006; IL-6 adjusted gPTSD IVW beta = 0.174, SE = 0.043, *p* = 5.2 × 10^−5^; WBC adjusted gPTSD IVW beta = 0.163, SE = 0.044, *p* = 0.0002) compared to the unadjusted gPTSD estimate.

It is also possible that significant MR associations were confounded by an unmeasured third factor with causal effects on both PTSD and the target phenotypes (i.e., correlated horizontal pleiotropy). To determine whether there was evidence for causation beyond what could be accounted for by correlated horizontal pleiotropy, we compared nested competing models. Results were considered consistent with a causal effect if the model with a causal effect parameter (causal model) provided a significantly better fit than the reduced model fit with only a shared effect parameter for correlated horizontal pleiotropy (sharing model). Our analyses supported causal effects of PTSD on autoimmune thyroid disease (beta = 0.11, 95% CI = [0.07–0.14]; causal versus sharing model *p* = 2.6 × 10^−3^) and CRP (beta = 0.05, 95% CI = [0.04–0.07]; causal versus sharing model *p* = 8.5 × 10^−8^) (Supplementary Table 7). We note that the causal effect estimates of these models were comparable in magnitude (overlapping 95% CIs) to the IVW estimates. In testing for the effects of genetically predicted phenotypes on PTSD (Supplementary Table 8), the causal model of CRP on PTSD did not substantially improve fit over the sharing model (causal versus sharing model *p* = 0.18), thus indicating no strong evidence of a causal effect.

## Discussion

In this most comprehensive study to date of potential causal relationships between PTSD and autoimmune disease based on their genetic underpinnings, an initial screening of their genetic overlap indicated broadly different genetic architectures. Relative to PTSD, a greater proportion of the phenotypic variation in numerous autoimmune diseases is accounted for by common genetic variation. Moreover, relatively few variants (from a few dozen to a few hundred) explain the majority of *h*^2^_SNP_ in autoimmune diseases, whereas relatively more variants (~10,000) are needed to explain the *h*^2^_SNP_ of PTSD. Despite these differences, we have identified shared genetic variation between PTSD and autoimmune diseases. Our results would suggest that this overlap is more nuanced than what is indicated by genetic correlation, such that a substantial fraction of variation influential to autoimmune diseases also influences PTSD. Thus, our results support hypotheses that shared underlying biology contributes to their epidemiologic associations [7–14]_._ We expect that detailed interrogation of shared loci [63] and systems [64] between PTSD and autoimmune diseases will provide better insights into their shared biology. Indeed, a recent familial coaggregation study [65] has identified five shared functional modules (potential molecular complexes [66]) between PTSD and autoimmune diseases, including signaling by G proteins/G protein complex receptors, an essential component of immune response [67]_._

We also leveraged genetic associations from GWAS to investigate the causal relationship between PTSD and autoimmune disease, as it will help determine clinical relevance of the recognition and treatment of PTSD in individuals living with primary disorders of the immune system [19] and provide directions for future mechanistic research. Our results support a putative causal effect of PTSD on autoimmune thyroid disease, corroborating findings from large prospective cohort studies suggesting that PTSD precedes the development of autoimmune thyroid disease [7–9]. In suggesting that the relationship is causal, our results strongly support the notion that clinical attention should be paid to thyroid health in those with PTSD symptoms [68]. However, given the broad phenotyping used for autoimmune thyroid disease, it is unclear whether our findings relate to specific types of autoimmune-related thyroid dysfunction (i.e., Grave’s or Hashimoto’s). Further research is needed to explore this question as more nuanced genetic instruments are generated. In terms of potential genetically regulated mechanisms linking PTSD to autoimmune thyroid disease, we considered the shared genetic signal between PTSD and inflammatory biomarkers that we have identified. However, adjusting for inflammatory biomarkers in multivariate MR did not substantially influence the positive association of PTSD with autoimmune thyroid disease. Thus, from our data alone, it is unclear what factors drive the putative causal association between PTSD and autoimmune thyroid disease. Prior studies have suggested some shared biological pathways between these conditions, including “signaling by G proteins/GPCRs,” “cilium assembly,” and “membrane trafficking” [65]. Additionally, our findings do not provide insight into PTSD’s potential pathophysiologic alterations on thyroid function through other mechanisms, such as through metabolic alterations [69] or effects on the central nervous system [70], making these future points of investigation.

We speculate on whether the influence of PTSD on autoimmune disease risk is mediated by the mechanisms considered to be highly relevant to PTSD pathophysiology. PTSD is associated with alterations in the hypothalamic-pituitary-adrenal (HPA)-axis [71], which plays a key role in stress response and also regulates the immune system [72]. Indeed, glucocorticoids released by activation of the HPA axis regulate a range of immune-related genes, including the expression of inflammatory cytokines [15]. In the context of our findings, dysregulation of the HPA-axis in PTSD may interfere with regulation of immune function by the HPA-axis, thereby leading to excessive inflammation [15]. In fact, impaired HPA responsiveness, such as found in PTSD, has demonstrated associations with other autoimmune and inflammatory diseases [73]. It is also important to note that certain aspects of HPA-axis dysregulation result from trauma exposure rather than PTSD [74]. This prompts the question of whether trauma exposure itself (or trauma of a particular type or timing [75]) is a driving force behind our observed associations. In considering other mediating mechanisms, Song et al. speculated that trauma-related lifestyle changes may influence autoimmune disease risk [8]. Ultimately, the clinical implications of our findings would be greatly enhanced if risk pathways were delineated. This delineation will inform whether the most relevant modalities to reduce the elevated risk of autoimmune disease development involve the treatment of PTSD itself, early interventions to reduce trauma exposure, or alterations to trauma-related lifestyle changes. One possible approach for a future investigation leveraging genomic data to estimate the contribution of these factors would be to conduct network MR analyses [76].

In assessing hypotheses that PTSD produces an inflammatory state [15], we observed a putative causal effect of PTSD on CRP. Contrary to our expectation of a bidirectional association based on prior work [26], we found no causal influence of immune/inflammatory biomarkers on PTSD. Rather, our results suggest that the previously reported causal effect of CRP on PTSD [26] was confounded by a (unmeasured) genetically determined factor. At first glance, this seems to conflict with existing epidemiologic evidence that elevated CRP precedes PTSD development [77]. However, we note that a genetically determined confounder could lead to elevated CRP before the development of PTSD. Thus, our results only argue against the hypothesized mechanistic role of CRP in PTSD [78], and do not weigh against hypotheses that inflammation causally influences PTSD [79]. With the increasing availability of genetic data, as with autoimmune disease, locus- and systems-level interrogations may help further validate this hypothesis or elucidate alternative mechanisms. Indeed, recent genomic studies converge to support the notion that there is systemic immune dysregulation in PTSD and importantly, highlight specific genes and pathways [16]. These will make excellent candidates to explore in future genomic studies of the overlap of PTSD, inflammation, and autoimmune disease.

In contrasting our findings with epidemiologic studies [7–9] of PTSD and autoimmune disease, these studies identified a wide spectrum of disease associations that we did not identify here. The effect sizes reported for these conditions varied, but altogether were relatively modest (hazard ratios and relative risks of ~2 or less). Thus, null association in our investigation may reflect power limitations of our analyses, rather than evidence that epidemiologic associations were confounded. In regard to power, of all autoimmune disease GWAS we examined, the autoimmune thyroid disease GWAS had the largest sample size and was thus likely the most powered. Similarly, a previous familial genetic and polygenic risk score based investigation of stress related disorders and autoimmune diseases [65] identified a significant association of PTSD with autoimmune thyroid disease, but yielded less conclusive evidence for other individual autoimmune diseases. The investigators hypothesized that the autoimmune thyroid disease association was identified due to its higher prevalence (and thus statistical power) relative to other autoimmune diseases, rather than effects specific to autoimmune thyroid disease [65]. Therefore, we speculate that significant associations with other autoimmune diseases may come to light as larger GWAS of autoimmune diseases are conducted.

Another factor that may have influenced our findings is the heterogeneity existing within autoimmune diseases [80]. If cases included in autoimmune disease GWAS are heterogeneous, effect estimates will not accurately capture risk, and this will in turn harm the reliability of MR. It will be useful to parse out whether PTSD influences risk of only particular subtypes of an autoimmune disease, as this can help identify more specific shared pathways. In regard to heterogeneity, we highlight how the age of onset of autoimmune disease reflects etiologic heterogeneity [81]. In the epidemiologic study by Song et al. [8], PTSD preceded the development of autoimmune disorders in adults that typically have an early age of onset (e.g., type 1 diabetes [81]). Thus, lack of corroborating findings here may reflect the need to specifically only use GWAS of adult-onset cases of autoimmune diseases in MR analyses. If it is the case that PTSD influences risk particularly in adult forms of autoimmune disease, this prompts the discussion of whether it will be important to consider PTSD status in the screening, management, and treatment of these diseases.

An additional notable challenge specific to conducting well-powered MR in this context is the usage of the HLA region as an instrumental variable. This region is highly relevant to many autoimmune diseases [82]. However, we excluded this region because of its high degree of pleiotropy and complicated LD structure, likely sacrificing statistical power as a consequence. Very detailed colocalization analysis [83] would need to be performed prior to integrating this region into PTSD MR analyses. Indeed, given its relevance to PTSD [84], the investigation of the HLA region should be a focal point of future research into the genetic overlap of PTSD and autoimmune diseases.

This study did not evaluate if effects observed for PTSD are specific to this psychiatric disorder. Autoimmune diseases are associated with other psychiatric disorders [23], which raises the possibility that shared variation between psychiatric disorders (general psychopathology) drives the observed associations. Individuals with psychiatric co-morbidities along with PTSD have elevated risk of developing autoimmune diseases relative to those with just PTSD [8], further suggesting the influence of other forms of psychopathology on autoimmune disease development. We also note the wide symptom overlap of PTSD with other psychiatric conditions, promoting the possibility that the observed associations arose from shared symptoms, rather than PTSD-specific symptomatology. Moreover, our definition of PTSD in the PGC-PTSD GWAS included participants with non-clinical assessments of PTSD, thus increasing the potential for misclassification generating non-PTSD-specific results. In considering mediating mechanisms such as HPA-axis dysregulation, we note how alterations in the HPA-axis are not specific to PTSD, and are present in other trauma-relevant mental disorders, e.g., bipolar disorder, borderline personality disorder [85], and depression (albeit with differences in manifestation [86]). In defense of the relevance of PTSD, epidemiologic evidence suggests that individuals with PTSD are at significantly higher risk of autoimmune disease than those afflicted by other mental health disorders [9]. We propose that future MR analyses of more homogenous PTSD symptom dimensions may help to distinguish risk. Namely, it would be interesting to see whether autoimmune disease risk is particularly elevated by the re-experiencing symptoms that are a hallmark of PTSD rather than symptoms that are shared with other forms of psychopathology [87].

While we performed several sensitivity analyses to assess the validity of our MR analyses, we highlight some potential limitations. First, the high polygenicity and low discoverability of PTSD pose challenges to the selection of valid SNP instruments for MR. Namely, the pathways by which implicated SNPs influence PTSD risk are not obvious, and thus it is not clear whether these SNPs could have confounding horizontal pleiotropic effects on autoimmune disease. Indeed, our results indicate heterogeneity across instruments for many autoimmune diseases, suggesting horizontal pleiotropy, albeit adjusting for this did not substantially alter results. In the future, the MR framework [88] could be used to help understand the causal pathways of risk SNPs, leading to the best possible selection of SNP instruments for PTSD. Second, there was potential for sample overlap between PTSD and a subset of the autoimmune GWAS (such as data originating from widely-used sources such as the UK BioBank), which can potentially bias two-sample MR analyses due to weak instrument bias. Recent work demonstrates that many two sample MR approaches can successfully be used for one-sample MR [89], a scenario of complete sample overlap where weak instrument bias is a primary concern. Importantly, our investigation used genome-wide significant variants from large GWAS, which demonstrate strong, replicable associations. Thus, our results are less likely to be influenced by the weak instrument bias that arises from using underpowered GWAS. Last, associations of PTSD with autoimmune and thyroid disease and CRP were still significant when we removed the UKBB sample.

In summary, shared genetics between PTSD and autoimmune diseases may underlie, in part, their epidemiologic associations. We also observed a putative causal effect of PTSD on autoimmune thyroid disease and CRP, consistent with epidemiologic evidence of stress- and trauma-related disorders predicting elevated systemic inflammation and onset of autoimmune disease [7–9, 85]. Together, these results highlight the nuanced links between PTSD, autoimmune disorders, and associated inflammatory signatures, and suggest the importance of targeting related pathways to protect against disease and disability.

### Supplementary information

Supplementary Figure 1

Supplementary Tables

## Data Availability

URLs for GWAS summary data used in this manuscript are provided in Supplementary Table 1.

## Code availability

Analysis code is available on GitHub (https://github.com/nievergeltlab/PTSD_MR).
