# Supplementary figures and images for: Effects of genetically predicted posttraumatic stress disorder on autoimmune phenotypes
Source: Transl Psychiatry. 2024 Apr 1;14:172. doi: 10.1038/s41398-024-02869-0 (PMC10984931; doi:10.1038/s41398-024-02869-0)

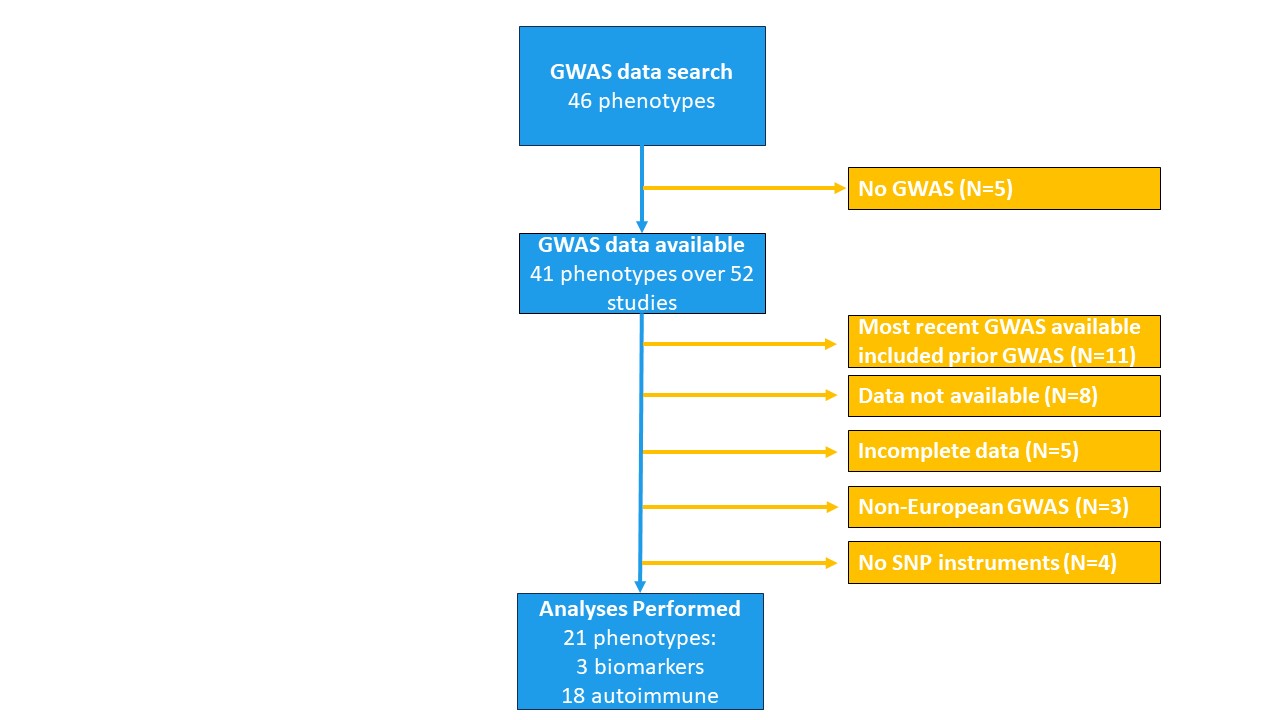

Supplement: Supplementary file 1 — Supplementary Figure 1 [file 41398_2024_2869_MOESM1_ESM.jpg]
